# "The Policy Dystopia Model": Implications for Health Advocates and Democratic Governance

**DOI:** 10.1371/journal.pmed.1002126

**Published:** 2016-09-20

**Authors:** Elizabeth A. Smith, Patricia A. McDaniel

**Affiliations:** Department of Social and Behavioral Sciences, University of California, San Francisco, San Francisco, California, United States of America

## Abstract

In this Perspective on the research article by Ulucanlar and colleagues, Elizabeth Smith and Patricia McDaniel discuss how industry opposition to regulation can undermine the public's overall confidence in government and science.

A new window on tobacco industry operations was opened first in 1994 when a few thousand secret tobacco industry documents were leaked to a tobacco control researcher and advocate [1] and later, in 1998, when millions more were released as part of the Master Settlement Agreement between the tobacco industry and the Attorneys General of 46 states [2]. The last 20-plus years of mining these documents, in addition to revealing telling details about sales and marketing tactics, has exposed numerous previously undetected tobacco industry activities. Chief among these was the creation of doubt about the dangers of tobacco use, accomplished by polluting the scientific record about the diseases caused by primary and secondhand smoke [3]. The tobacco industry paid researchers, developed studies, and even created its own organizations and journals in order to create the impression that there was no conclusive evidence that its products killed millions of people [4,5].

In muddying the science demonstrating smoking’s lethal effects, the tobacco industry’s goal is generally to avoid strong regulation that might reduce sales. But, as Ulucanlar and colleagues make clear in this issue of *PLOS Medicine*, an assault on science by the tobacco industry is only one piece of a larger pattern of strategies to ensure that policies it opposes are rejected, delayed, watered down, prohibited, reversed, or otherwise consigned to failure [6]. These strategies include a range of arguments promulgated by a variety of voices (for example, having a representative of law enforcement claim that a proposed policy will increase illicit trade and a social reformer claim that it is regressive) as well as direct actions such as creating and managing coalitions, lobbying, and litigation. Such approaches have effects far beyond the thwarting of tobacco control measures.

## Doubt about Science

One side effect of the tobacco industry’s false scientific narrative was the creation of doubt about science in general. News organizations frequently report uncritically on scientific “controversies” about tobacco, leading some who are unable to assess the quality of the studies to conclude that a “scientific” study can be found to support any position [7]. Other industries that stand to profit from this kind of confusion may adopt the approach of manufacturing doubt about the health or environmental impacts of their products. It has been suggested, for example, that Coca-Cola funded the Global Energy Balance Network of scientists to promote the unsupported view that exercise is more important than diet in maintaining a healthy weight, thus creating doubt about the role of sugary drinks in the obesity epidemic [8]. Similarly, the fossil fuel industry has followed the tobacco industry’s playbook in casting doubt on the scientific consensus regarding global warming [9].

## Doubt about Democracy

The strategies outlined by Ulucanlar and colleagues also threaten the role of government in safeguarding public health. Sound policies may be rejected by legislative bodies or watered down to the point that they do not solve the problem at hand. For instance, after tobacco industry lobbying, strong, simple, clean indoor air legislation has been riddled with exceptions that seem arbitrary and prevent the public from expecting public spaces to be smoke-free [10]. Alternatively, once a law is passed, the industry may implement a campaign of covert or overt noncompliance. For example, tax increases may be undermined through industry-countenanced cigarette smuggling [11]. In these cases, citizens may draw the conclusion that policy change and, indeed, government itself are ineffective. This distrust leaves a gap that a variety of industries are more than willing to fill with their own voluntary programs, which have largely been shown to be ineffective at improving public health and instead improve the status of their sponsors [12,13]. In the worst-case scenario, this creates a downward spiral in which government increasingly loses power and, as a result, the support of citizens. Citizens become disengaged or angry, leaving government without the needed mandate to act for public health against industry interests, and the cycle continues. The analysis that Ulucanlar and colleagues provide raises the concern that industry policy strategy can ultimately undermine democratic government.

Advocates concerned about policy approaches to any health problem would be well advised to pay as much attention to alliances, argumentation, and policy levers as the industry has. Advocates should also keep an eye on this bigger picture of supporting democratic engagement more broadly. The history of tobacco control is one of success that has come from the bottom up, from policy innovations at the local level only later adopted by state and national governments. It is at the local level that people can most readily feel the effectiveness and empowerment of democratic government. Supporting this kind of advocacy strengthens the policy process as well as specific policy outcomes. In this way, advocates can create a compelling affirmative policy narrative to counter the industry’s dystopian vision.
